# Learning curve for laparoscopic Heller myotomy and Dor fundoplication for achalasia

**DOI:** 10.1371/journal.pone.0180515

**Published:** 2017-07-07

**Authors:** Fumiaki Yano, Nobuo Omura, Kazuto Tsuboi, Masato Hoshino, Seryung Yamamoto, Shunsuke Akimoto, Takahiro Masuda, Hideyuki Kashiwagi, Katsuhiko Yanaga

**Affiliations:** From the Department of Surgery, The Jikei University School of Medicine, Tokyo, Japan; Saga University Hospital, JAPAN

## Abstract

**Purpose:**

Although laparoscopic Heller myotomy and Dor fundoplication (LHD) is widely performed to address achalasia, little is known about the learning curve for this technique. We assessed the learning curve for performing LHD.

**Methods:**

Of the 514 cases with LHD performed between August 1994 and March 2016, the surgical outcomes of 463 cases were evaluated after excluding 50 cases with reduced port surgery and one case with the simultaneous performance of laparoscopic distal partial gastrectomy. A receiver operating characteristic (ROC) curve analysis was used to identify the cut-off value for the number of surgical experiences necessary to become proficient with LHD, which was defined as the completion of the learning curve.

**Results:**

We defined the completion of the learning curve when the following 3 conditions were satisfied. 1) The operation time was less than 165 minutes. 2) There was no blood loss. 3) There was no intraoperative complication. In order to establish the appropriate number of surgical experiences required to complete the learning curve, the cut-off value was evaluated by using a ROC curve (AUC 0.717, p < 0.001). Finally, we identified the cut-off value as 16 surgical cases (sensitivity 0.706, specificity 0.646).

**Conclusion:**

Learning curve seems to complete after performing 16 cases.

## Introduction

Laparoscopic surgery is less invasive than open abdominal surgery, and has come into broad use because it is excellent from the viewpoint of cosmetic results [1]. Additionally, in deep tissue regions such as the esophageal hiatus, mediastinum, and pelvis, laparoscopy can provide superior visibility compared with the naked eyes [2]. However, one disadvantage is that tactile sensation is minimized thorough instruments. In spite of reports of serious complications and patient death [3, 4], laparoscopic surgery continue to increase each year. The Endoscopic Surgical Skill Qualification System was established in Japan to promote safer and more appropriate laparoscopic operations [5]. Becoming certified as a qualified surgeon is a major goal for today's gastrointestinal surgeons in Japan.

Laparoscopic Heller myotomy and Dor fundoplication (LHD) is a procedure that was developed as a surgical treatment for achalasia. Although this procedure does not require in gastrointestinal anastomosis, LHD involves exposure and detachment of the esophageal hiatus, dissection of the short gastric arteries/veins, an incision at the external mucous membrane of the esophageal muscle layer, and suture/ligation procedures between the gastric fundus and the esophagus as part of gastroesophageal reflux prevention procedures, encompassing most of the basic procedures of laparoscopic surgery. This type of operation is also relatively prone to causing intraoperative complications such as damage to the esophagus or gastric mucosa [6]. As such, LHD is considered a procedure appropriate for surgeons aiming to become certified as qualified endoscopic surgeons. However, esophageal achalasia is a rare condition, with a morbidity rate of 1–2 cases/100,000 persons [7]. Based on the results of our literature search, while there are several reports regarding per-oral endoscopic myotomy (POEM) and the learning curve for performing Heller myotomy [8–16], there have been no reports concerning LHD in the treatment of achalasia. Therefore, we assessed the learning curve for performing LHD. The aim of this study was to investigate the learning curve for not only Heller myotomy but also Dor fundoplication for achalasia.

## Material and methods

### Patient population

Data on all patients undergoing LHD at our department are stored in a prospectively maintained database. After approval from the Jikei University School of Medicine Institutional Review Board (28–059, 8302), the data were investigated and retrospectively reviewed. The ethical committee has accepted the database and given permission for using it for research purposes. Therefore, our IRB waived the need for consent. This is the retrospective study and the data were processed anonymously, so no consent was given. Instead of the consent, the information was posted in our hospital about the current study and all patients received the right to opt out from study.

Of the 514 cases in which LHD was conducted between August 1994 and March 2016, the surgical outcomes of 463 cases were evaluated after excluding 50 cases for reduced port surgery and one case for simultaneous laparoscopic distal partial gastrectomy (S1 File).

### Patient backgrounds and conditions

We examined patient age, sex, and disease period as patient background data. In addition, in accordance with the Japanese esophageal achalasia treatment guidelines [17], we assessed the type and degree of dilatation as well as the maximum esophageal transverse diameter.

### Evaluation of surgical performance

We surveyed the attending surgeons in each case and investigated the surgeons specific number of LHD operations. To evaluate surgical results, operation time, amount of blood loss, presence of intraoperative complications (esophageal or gastric mucosal injuries and/or other organs injuries), presence of postoperative reflux esophagitis, and postoperative satisfaction score were assessed. Satisfaction score was obtained via a questionnaire administered ≥ 3 months after the operations; the degree of improvement relative to preoperative symptoms was defined using the following 5 grades: 1: Poor; 2: Fair; 3: Moderate; 4: Good; 5: Excellent.

### Relationship between number of LHD cases experienced by the surgeon and surgical performance

The 5 evaluation items of surgery time, amount of blood loss, intraoperative complications, occurrence of postoperative reflux esophagitis, and satisfaction score were used to examine the impact of the number of LHD experiences on the surgical results. We analyzed the presence or absence of correlations with the number of LHD cases experienced by the surgeon, using Spearman’s rank correlation test. Next, we extracted parameters correlated with the number of LHD cases experienced.

### Assessment of cut-off values in creating a learning curve

For each parameter in relation to the number of LHD cases experienced by the surgeon, we designated each target and searched for the number of surgical experiences (cut-off value) necessary to achieve such a target. Specifically, a Receiver Operating Characteristics (ROC) curve analysis was performed to identify the cut-off value for the number of surgical experiences defined as exceeding the learning curve.

### Assessment of cut-off value credibility

After cut-off values were obtained, the samples were divided into 2 groups, and the credibility of the cut-off values was examined by comparing patient backgrounds and conditions with surgical results.

## Statistical analysis

All statistical analyses were performed using SPSS version 22.0.0.0 (Armonk, NY, USA). Medians with interquartile ranges (IQR) are expressed for continuous variables. Chi-square test was used to compare categorical variables. Mann-Whitney’s U test were used to compare continuous variables. A ROC curve analysis was used to identify the cut-off value of the surgical experience number which was defined as the completion of the learning curve. Relationships between operation time, amount of blood loss, intraoperative complications, postoperative reflux esophagitis, and satisfaction scores, as reflected in questionnaire responses, were assessed using a Spearman’s rank correlation test. The level of significance was set at a *p* value <0.05.

## Results

### Patients’ characteristics and surgical outcomes

Median patient age was 45 years, and 208 were females (44.9%), while the median disease period was 60 months. The morphology of the lower esophageal segment was as follows: straight type in 375 patients (81.9%), sigmoid type in 65 patients (14.2%), and advanced sigmoid type in 18 patients (3.9%). The median value for the maximum transverse diameter (d) of the esophagus was 5.0 cm, where d <3.5cm was defined as grade I (n = 54, 11.8%); 3.5 ≤ d < 6.0 cm, as grade II (n = 265, 58%); and 6.0 cm ≤ das grade III (n = 138, 30.2%). The median values for total operation time and amount of blood loss were 165 minutes and 0 mL (uncountable), respectively. Intraoperative complications occurred in 81 patients (17.5%) and postoperative reflux esophagitis occurred in 51 patients (15.5%). The median postoperative satisfaction score was 5 (Table 1).

**Table 1:** Patients’ characteristics and surgical outcomes.

Age (years)^†^	45 (33–57)
Sex (M/F)	255/208
Duration of disease (mo) ^†^	60 (24–120)
Morphologic type (St/Sg/aSg/unknown)	375/65/18/5
Grade of dilatation (I/II/III/unknown)	54/265/138/6
Maximum transverse diameter (cm) ^†^	5.0 (4.4–6.2)
Operation time (min) ^†^	165 (139–192.5)
Blood loss (ml) ^†^	0 (0–0)
Intraoperative complications (Yes/No)	81/382
Postoperative reflux esophagitis (Yes/No/unknown)	51/277/135
Satisfaction score (1–5) ^†^	5 (5–5)

^†^; median (interquartile range: IQR), mo; months, St; straight type, Sg; sigmoid type

aSg; advanced sigmoid type. Satisfaction score (1; Poor, 2; Fair, 3; Moderate, 4; Good, 5; Excellent).

### Surgeons

Twenty-five surgeons performed surgery on 463 patients, and the average number of cases per surgeon was 18.5 (1–63) cases. Fifteen of the 25 surgeons (60%) were certified as qualified surgeons under the Endoscopic Surgical Skill Qualification System as of November 2016. In addition, 9 surgeons (36%) were certified for esophageal surgery, and 6 (24%) were proficient in performing LHD surgery.

### Correlations between number of LHD cases experienced by the surgeon and surgical performance

**Fig 1:**
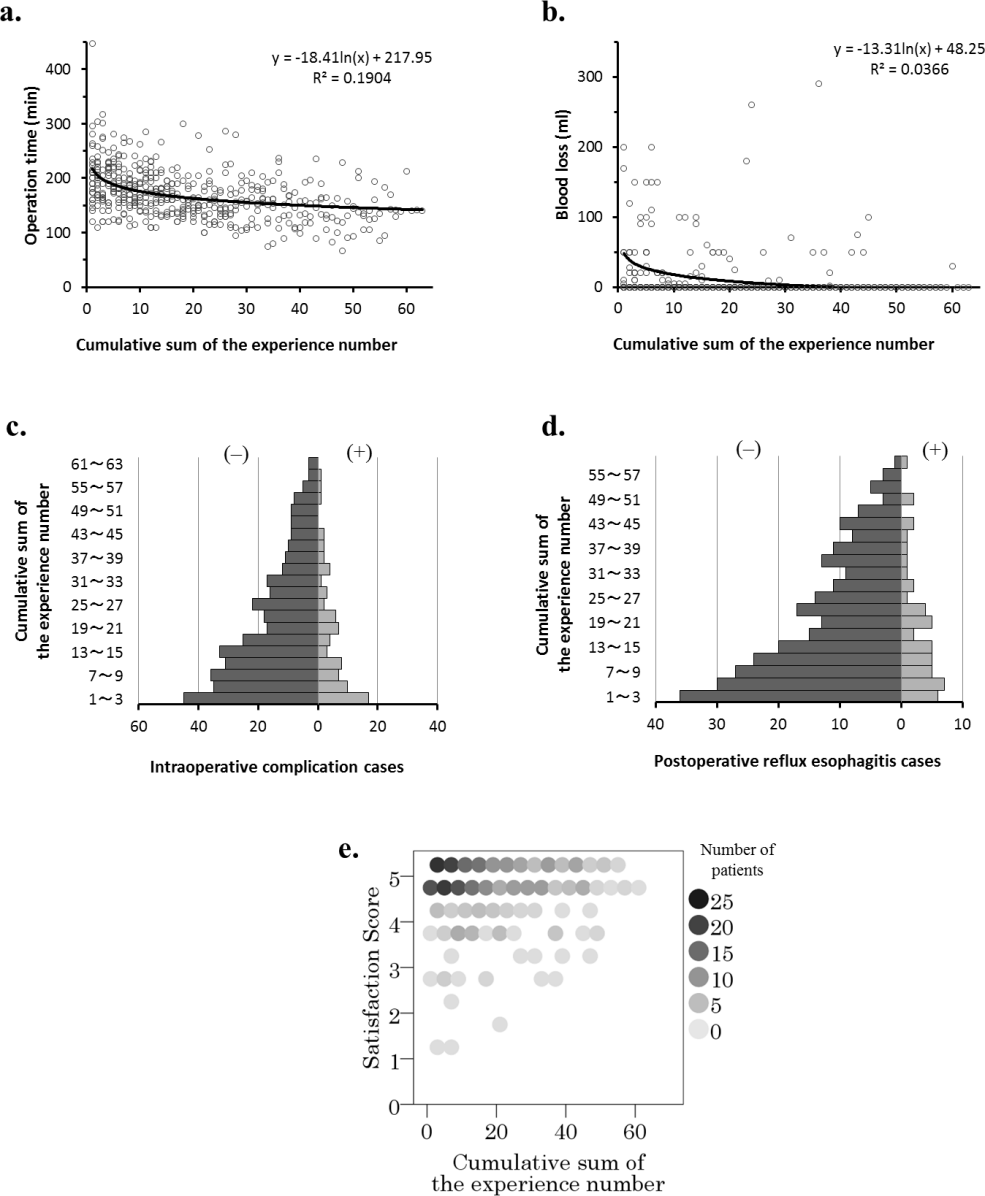
Assessment of correlations between surgical experiences and each surgical performance evaluation parameter. **A.** Correlation with surgery time: r_s_ = -0.446, p<0.001 **B.** Correlation with blood loss: r_s_ = -0.206, p < 0.001 **C.** Correlation with intraoperative accident occurrences (missing values: 8): r_s_ = -0.106, p = 0.023 **D.** Correlation with occurrences of postoperative reflux esophagitis (missing values: 135): r_s_ = -0.032, p = 0.567 **E.** Correlation with satisfaction score: r_s_ = 0.019, p = 0.719.

1. Correlation with operation time (Fig 1A)
  There was a significant correlation between the operation time and the cumulative sum of the number of achalasia surgeries performed by the surgeon (r_s_ = −0.446, p < 0.001).
2. Correlation with amount of blood loss (Fig 1B)
  There was a significant correlation between blood loss and the cumulative sum of experiences (r_s_ = −0.206, p<0.001).
3. Correlation with intraoperative complication occurrences (Fig 1C)
  There was a significant correlation between intraoperative complication and the cumulative sum of experiences (r_s_ = −0.106, p = 0.023).
4. Correlation with postoperative reflux esophagitis cases (Fig 1D)
  There was no significant correlation between postoperative reflux esophagitis and the cumulative sum of experiences (r_s_ = −0.032, p = 0.567).
5. Correlation with satisfaction score (Fig 1E)

There was no significant correlation between satisfaction score and the cumulative sum of experiences (r_s_ = 0.019, p = 0.719).

### Assessment of cut-off values in creating a learning curve

Based on the above correlations, operation time, amount of blood loss, and intraoperative complications were selected and examined as parameters for the assessment of surgical performance.

**Fig 2:**
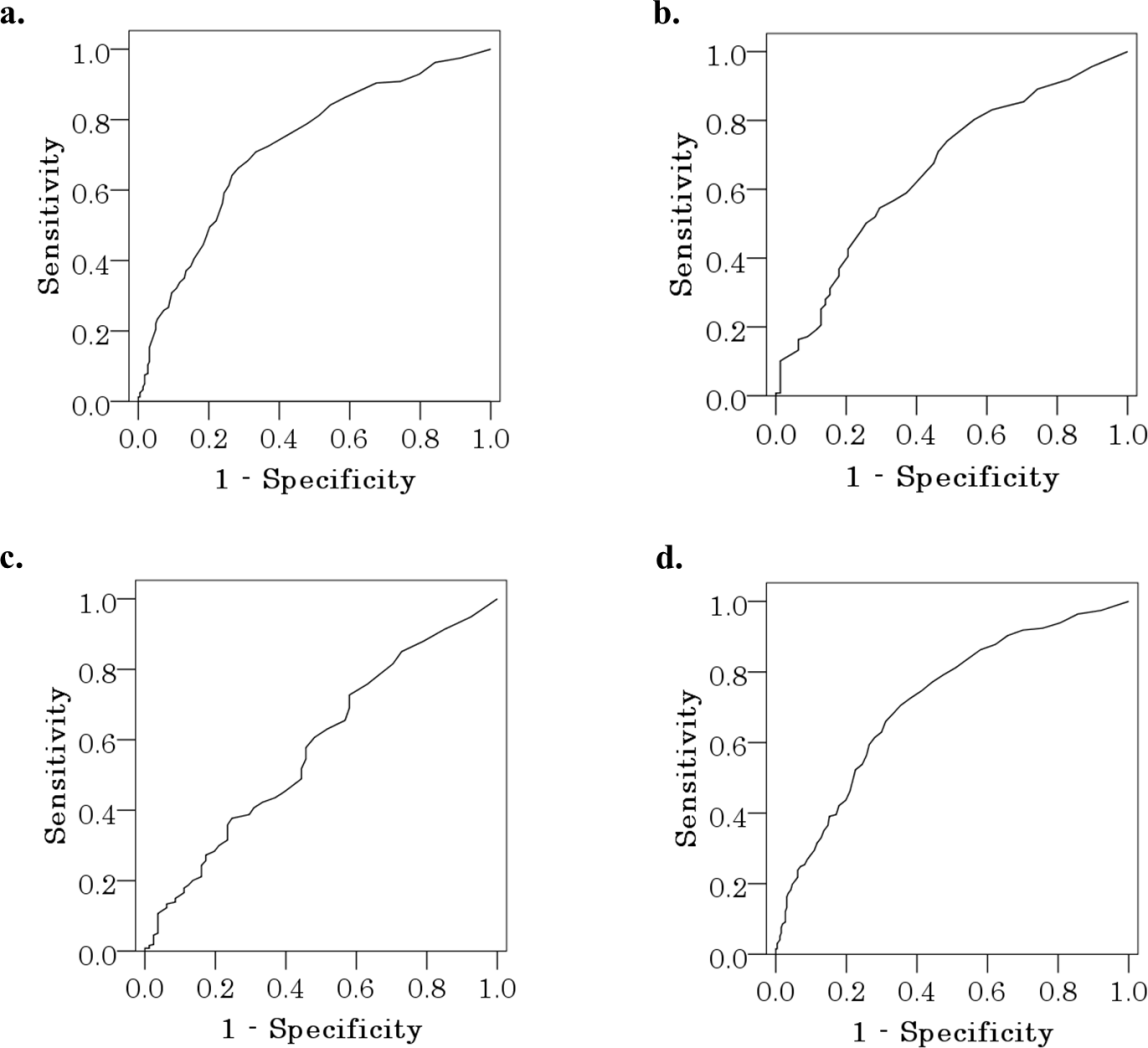
ROC curves for each parameter. **A.** When a target operation time of under 165 minutes was designated, AUC was 0.724 and *p*<0.001. **B.** When a target of no blood loss was designated, AUC was 0.658 and *p =* 0.001. **C.** When a goal of no intraoperative accidents was designated, AUC was 0.580 and *p* = 0.024. **D.** When a operation time of under 165 minutes, no blood loss, and no intraoperative accidents were designated as achievement targets, AUC was 0.717, *p*<0.001, and cut-off value detection was sufficiently precise.

1. Operation time
  As the median operation time was 165 minutes, when this length of time was designated as the target, the ROC curve exhibited an area under the curve (AUC) of 0.724, *p*<0.001, and the cut-off value was 17 surgical cases (Fig 2A).
2. Amount of blood loss
  With no blood loss as the target, the ROC curve exhibited an AUC of 0.658, *p*<0.001, and the cut-off value was 13 surgical cases (Fig 2B).
3. Intraoperative complications
  With no intraoperative complications as the target, the ROC curve exhibited an AUC of 0.580, *p* = 0.024, and the cut-off value was 15 surgical cases (Fig 2C).
4. Cut-off values based on all parameters

When a total operation time of 165 minutes, no blood loss, and no intraoperative complications were designated as the target for LHD surgery performance, the ROC curve exhibited an AUC of 0.717 (*p*<0.001), and the cut-off value was 16 surgical cases (Fig 2D, sensitivity 0.706, specificity 0.646).

### Assessment of cut-off value credibility

**Fig 3:**
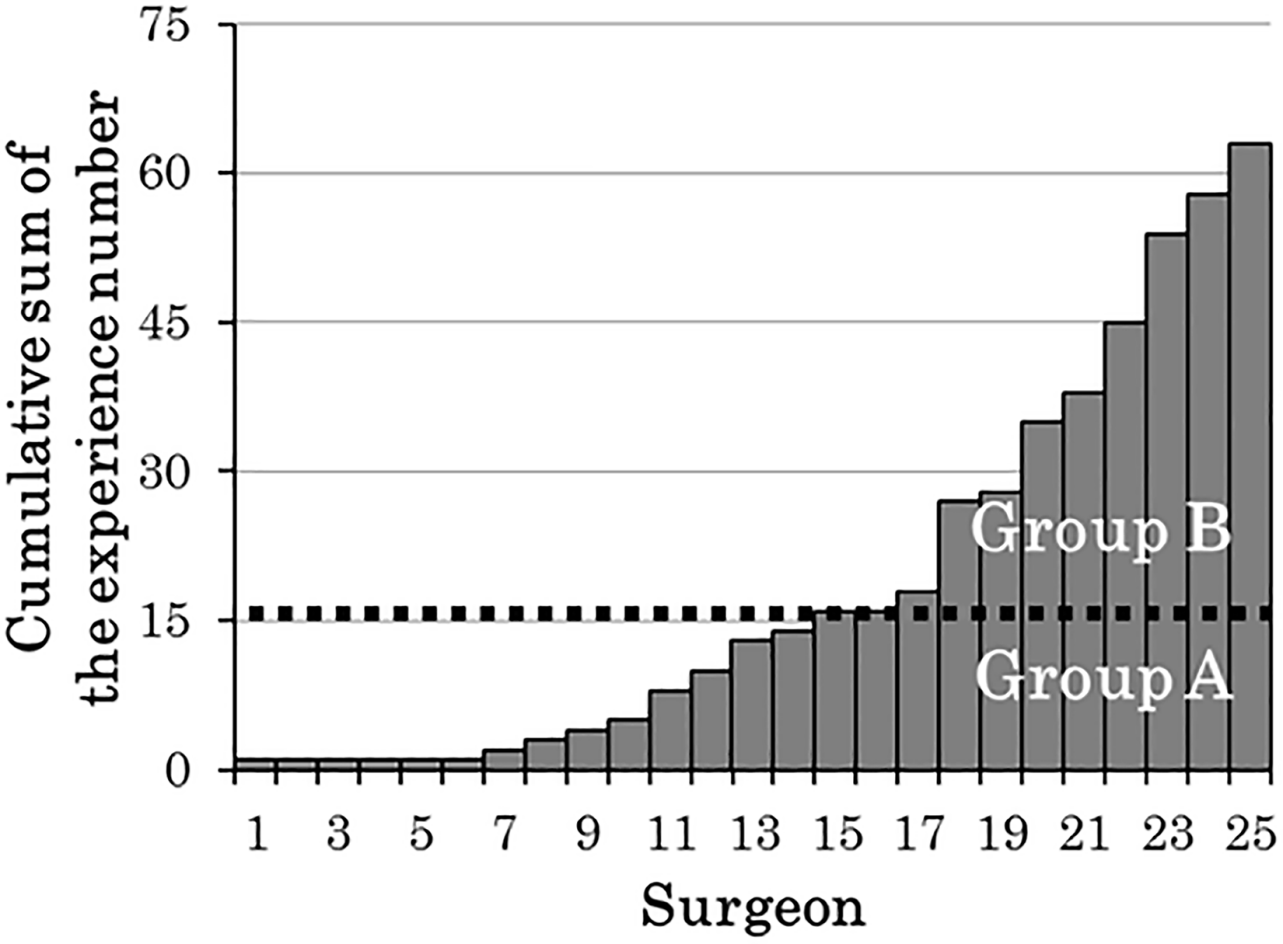
Number of surgical experiences per surgeon.

As the cut-off value was 16 cases, patient backgrounds, preoperative pathophysiology, and surgical results were compared and examined by dividing the surgeons into two groups: surgeons with surgical experience of less than 16 cases (Group A, n = 230) and surgeons who had experienced 16 or more cases (Group B, n = 233) (Table 2, Fig 3). There was no significant difference between any of the median values for age, sex, or disease period (p = 0.287, 0.754, and 0.539, respectively). A significant difference was observed regarding the morphology of the lower esophageal segment (*p* = 0.038), but no difference was observed between the grade of esophageal dilatation and the maximum transverse diameter (*p* = 0.089 and 0.909, respectively). For surgical performance, total surgery time was significantly shorter in Group B (*p*<0.001), and total blood loss was also significantly lower (*p*<0.001). However, no significant difference was observed between the groups with regard to the number of intraoperative complications, occurrences of postoperative reflux esophagitis, or satisfaction score (*p* = 0.225, 0.475, and 0.730, respectively). The incidence of clarifying surgery target (total surgery time of 165 minutes, no blood loss, and no intraoperative complications) was significantly higher in Group B (Group A: 26.0%; Group B: 60% (*p<*0.001)).

**Table 2:** Assessment of the credibility of the 16 cut-off values.

	Group A (n = 230)	Group B (n = 233)	*p*-value
Age (years)^†^	43 (33–57)	46 (34–58.5)	0.287*
Sex (M:F)	125/105	130/103	0.754**
Duration of disease (mo)^†^	60 (28–120)	60 (24–120)	0.539*
Morphologic type (St:Sg:aSg:unknown)	195/30/4/1	180/35/14/4	0.038**
Grade of dilatation (I/II/III/unknown)	20/141/67/2	34/124/71/4	0.089**
Maximum transverse diameter (cm) ^†^	50 (42–60)	50 (40–61.5)	0.909*
Operation time (min) ^†^	180 (159–210)	148 (125.5–170.5)	<0.001*
Blood loss (ml) ^†^	0 (0–0)	0 (0–0)	<0.001*
Intraoperative complications (Yes/No/unknown)	45/180/5	36/194/3	0.255**
Postoperative reflux esophagitis (Yes/No/unknown)	28/137/65	23/140/70	0.475**
Satisfaction score (1–5) ^†^	5 (5–5)	5 (5–5)	0.730*
Achieved goals (Yes/No/unknown)	58/166/6	139/91/3	<0.001**

^†^; median (interquartile range: IQR), mo; months, St; straight type, Sg; sigmoid type, aSg; advanced sigmoid type

*; Mann-Whitney’s U test

**; Chi-square test

## Discussion

There have been reports pertaining to learning curves in various types of laparoscopic surgery, such as laparoscopic distal pancreatectomy and laparoscopic partial nephrectomy. Ricci et al. [18] examined 32 cases involving laparoscopic distal pancreatectomy performed by a single surgeon (pancreatic surgeon), with the primary endpoint being operation time and the secondary endpoint being the conversion to laparotomy, rate of complication, mortality rate, postoperative hospital stay, and number of unscheduled splenectomies. The ROC curve is used as a statistical method to calculate the cut-off value for the learning curve, as was conducted in the current study. The results indicated that conversion to laparotomy, incidence of complications, mortality rate, and postoperative hospital stay did not affect the surgeons’ ability to overcome the learning curve, and unscheduled splenectomies were performed relatively earlier than the completion of the learning curve. Based on these observations, the cut-off value was concluded to be approximately 17 surgical cases. However, Osaka et al. [19] evaluated 63 laparoscopic partial nephrectomies performed by one surgeon to address clinical T1a renal masses. To assess the results of surgery, predictive factors for achieving the designated performance targets were evaluated using three parameters: ischemia time of 25 minutes or less, negative margin, and no intraoperative complications. These 3 targets were met in 39 of 63 cases. Tumor size and the distance from the urinary tract were correlated with achievement of the 3 targets. Moreover, if separated by a learning curve of 30 cases, they concluded that the size of the tumor and the learning curve of the operator were predictors for achieving the 3 targets because the difference upon achievement of the targets was significant.

LHD to address achalasia is defined as laparoscopic surgery involving exposure and detachment of tissue proximal to the esophageal hiatus, dissection of short gastric arteries/veins, an incision at the external mucous membrane of the esophageal muscle layer, and suture/ligation procedures between the gastric fundus and the esophagus as part of gastroesophageal reflux prevention procedures; many procedures are necessary to become proficient with this technique. Although achalasia is a suitable condition for surgeons seeking technical certification, one disadvantage is that its incidence rate is low, manifesting in 1-2/100,000 patients [7]. Our facility is a high-volume center in Japan and more than 500 patients have undergone surgery by us. According to the case data compiled by the Japanese Society for Endoscopic Surgery, 1,876 LHDs have been performed between 1993 and 2015 [20], and 526 of these were performed at our facility during this period, approximately 30% of all LHDs performed in Japan. Accordingly, we believe that we have compiled a sufficient number of cases for assessing the learning curve.

The Endoscopic Surgical Skill Qualification System framework for accrediting qualified surgeons was established by the Japanese Society for Endoscopic Surgery in 2004 and the pass rate in the field of general and gastrointestinal surgery was 37% (1,606/4,387) over a period of 12 years by 2015. Among the candidates, the pass rate in the subfield of esophageal surgery ranges between 15–75% each year with the average of 41% (74/179) (http://www.jses.or.jp/member/gijutsuninteisinsakekka.html). As our facility currently employs 9 surgeons who have successfully obtained accreditation in the area of esophageal surgery, our facility retains 12% (9/74) of the surgeons holding this qualification throughout Japan.

Here, we describe the process that young surgeons undergo into becoming an LHD operator. To prevent medical errors in endoscopic surgeries, the Endoscopic Surgery Training Course Steering Committee was established as the internal certification system for operator qualification in our facility in April 2004. The clinical departments of gastrointestinal surgery, hepato-biliary-pancreatic surgery, thoracic surgery, pediatric surgery, obstetrics and gynecology, and urology participate in the system. Surgeons who are transferred to our facility can participate in endoscopic surgeries during a 6-month grace period but are required to attend lectures as part of a basic course and pass a written examination within this period. Those who pass the examination can participate in surgeries, but only within a certain scope. If they pass the practical examination in a dry laboratory in the next step, they are then allowed to handle forceps. After passing the practical examination in an animal laboratory using pigs and participating in more than 20 LHD operations, they are finally allowed to become an operator under the supervision of the attending surgeon.

By searching the PubMed database for reports as of November 28, 2016 using the key words “achalasia” and “learning curve”, we identified 2 reports concerning learning curves, excluding reports on POEM related to Heller myotomies to treat achalasia. Bloomston et al. [12] described a learning curve based on the experiences of 78 surgeons with Heller myotomies performed thoracoscopically or laparoscopically. This study divided the surgeons into an initial stage group with the first 25 cases, a mid-stage group with the second 25 cases, and a late stage group with 28 cases. The groups were evaluated based on the clinical outcome factors; number of inpatient days, number of intraoperative complications, rate of laparotomy transitions, and postoperative symptom improvement rate. Regarding intraoperative complications, the incidence was 20% in the initial group, 8% in the mid-stage group, and 12% in the late stage group, which were not statistically different. In addition, 3 cases were converted to laparotomy, all in the initial stage groups (*p*<0.05). Subsequently, every 10 consecutive cases were examined separately, and the number of inpatient days significantly decreased from the 20th case onwards (*p*<0.01). The rate of postoperative symptom improvement was also found to be significantly higher after the 20th case (80% vs. 96%, *p*<0.05), suggesting that the learning curve was roughly 20 cases of experience. In another report, Grotenhuis et al. [16] examined 186 treatment-naive surgical patients who underwent laparoscopic cardiomyotomy, and conducted the following 3 assessments: 1) evaluating cases chronologically without distinguishing by surgeon background (institutional experience); 2) evaluating each supervising physician in chronological order (individual experience); and 3) comparing supervisors and consultants (consultants vs. trainees). One hundred and forty-four cases were assigned to 5 supervisors, and the remaining 42 cases were handled by trainees. Regarding institutional experience 1), surgery time was significantly shorter between the first 10 cases and those afterwards (149 min. vs. 81 min., *p*<0.01), and conversion to laparotomy were higher in the first 20 cases at 20–30%, later falling to 0–2% with minimal variations (*p*<0.01). Intraoperative complications, degree of satisfaction, degree of improvement of dysphasia, and re-surgery rate were not affected by surgical experience. 2) When examining the learning curve for cases 1–40 for each supervising physician, the surgery time of cases 31–40 was significantly shorter than that of cases 1–10 (54 min. vs. 91 min., *p*<0.01). The rate of transition to laparotomy was 6% for the first 20 cases and 0% thereafter. Intraoperative complications, degree of satisfaction, degree of improvement of dysphasia, and re-do surgery rate were not affected by surgical experience. 3) When comparing supervisors and trainees, a significant difference was observed with regard to surgery time only (79 min. vs. 93 min., *p*<0.01), and no differences were observed between other results. They concluded from these observations that the institutional and individual learning curves were 20 and 10 case experiences, respectively, and there was no difference in surgical results between supervisors and trainees.

One issue regarding the examination method used by Bloomston et al. [12] is that the learning curve for an entire institution was obtained rather than the individual learning curves. In the report by Grotenhuis et al. [16], although individual learning curves were sought, 42 cases handled by trainees were excluded, and the goal was to compare the results of groups of 10 cases from the total of 144 surgeries performed by supervisors only. Further, as neither report had designated targets, the actual meaning of the learning curve remains ambiguous. As such, we clarified the goals to be achieved clinically and examined how many goals were achieved using the ROC curve. In addition, we targeted 463 patients who underwent surgery by 25 surgeons including trainees, and assessed the surgeons handling each case as well as the number of LHDs performed by each surgeon.

In order to designate performance targets, we selected 5 assessment metrics: operation time, amount of blood loss, number of intraoperative complications, satisfaction score and occurrence of postoperative reflux esophagitis, which are known general indicators of achalasia surgical performance [21, 22]. Correlations with the number of surgical experiences were evaluated using the Spearman’s rank correlation test with respect to these 5 metrics. Correlations were observed with respect to operation time, amount of blood loss, and number of intraoperative complications. It is easy to understand how operation time would be shortened, the amount of bleeding would be reduced, and the number of intraoperative complications would be decreased depending on the number of cases experienced previously by the surgeon. However, the reason satisfaction score and the incidence of postoperative reflux esophagitis are not affected by the number of surgical experiences is believed to be because supervising surgeons instruct surgeons so as to control the length of Heller myotomies and antireflux surgeries and the proper execution of Dor fundoplication. In addition, number of surgeries experienced has been highlighted as a risk factor for mucosal damage during LHD [6, 23]. The correlation between the number of surgeries experienced and number of intraoperative complications observed during this study supports this assertion. By using the 3 performance targets of operation time of 165 minutes or less, no blood loss, and no intraoperative complications, the ROC AUC of 0.717 could be determined and the cut-off value could be calculated as 16 cases.

Therefore, we further examined the credibility of the cut-off value of 16 case experiences. It was believed that there was a significant difference between groups A and B regarding preoperative histopathological type because of a large number of advanced sigmoid type cases in which the disease state improved when handled by more experienced surgeons in Group B. Operation time was significantly shorter in Group B, and the amount of blood loss was also smaller, but no significant difference was observed with respect to the incidence of intraoperative complications. The most common complication that occurs during LHD is damage to the esophagus or to the gastric mucosa [24]. Although mucosal damage is easily caused by momentary electrical discharge from electric scalpels or contact with sharp forceps, recommendations and advice from supervising surgeons urging frequent reduction in electrical transmission time and refraining from applying cricoid pressure at the esophageal muscle layer incision site are believed to be effective. However, the 60% (139/230) success rate of Group B, which was comprised of surgeons with experience handling numerous cases involving advanced conditions, was significantly higher than the rate of 26% (58/224) achieved by Group A. Based on these results, we believe that a cut-off value experience with of 16 cases is valid.

The present study has two main limitations.

First, there was a potential bias in the use of certain surgical procedures since it was a single-center, retrospective study. However, as explained above, our facility performs approximately 30% of the total number of cases of LHD in Japan and, despite it being a single center study, we believed that the number of patients included is sufficient to determine the learning curve for the procedure.

Second, the learning curves were determined solely based on the results of LHD by the 25 surgeons included in this study. There were also differences between the surgeons regarding the length of their general experience as physicians; however, we did not consider experience with operations other than LHD. While this limitation presents a problem for determining the learning curve, we did not believe that it was possible to consider each individual surgeon's total experience with all operations performed during their career. Nevertheless, LHD involves separation from surrounding tissue, the dissection of blood vessels, and suture ligation, which are the basic techniques of laparoscopic surgery. Therefore, we do not believe that it is meaningless to determine the learning curve on the basis of the experience with this surgical method alone.

## Conclusion

Through this study, we estimated the learning curve of laparoscopic Heller myotomy and Dor fundoplication for achalasia can be plateaued by performing 16 cases.

## Supporting information

S1 File The database of laparoscopic Heller-Dor procedure. 463 cases of laparoscopic Heller-Dor procedures were included in the present study.
(PDF)

## Abbreviations

LHD: laparoscopic Heller myotomy and Dor fundoplication
ROC: receiver operating characteristic
POEM: per-oral endoscopic myotomy
IQR: interquartile ranges
AUC: area under the curve
