# Supplementary material for: Learning curve for laparoscopic Heller myotomy and Dor fundoplication for achalasia
Source: PLoS One. 2017 Jul 7;12(7):e0180515. doi: 10.1371/journal.pone.0180515 (PMC5501549; doi:10.1371/journal.pone.0180515)
Supplement: S1 File — 463 cases of laparoscopic Heller-Dor procedures were included in the present study. (PDF) [file pone.0180515.s001.pdf]

Group(1=1−16srgical experiences,2=17−63surgical experiences)	number of surgical experiences in each surgeon	patient identity code	age	sex(m=1,f=2)	duration of disease (mo)	morphologic type (St=1,Sg=2,aSg=3)	grade of dilatation (I=1,II=2,III=3)
1	1	100001	26	1	6	1	2
1	1	100002	50	1	60	1	2
1	1	100003	45	2	156	1	3
1	1	100004	46	2	13	1	1
1	1	100005	47	1	360	1	3
1	1	100006	38	2	60	1	3
1	1	100007	47	2	120	1	2
1	1	100008	63	1	180	1	2
1	1	100009	32	2	72	1	2
1	1	100010	25	1	24	1	2
1	1	100011	63	2	84	1	2
1	1	100012	32	2	12	1	1
1	1	100013	43	2	48	1	2
1	1	100014	32	2	36	1	2
1	1	100015	60	2	132	2	2
1	1	100016	64	2	36	1	2
1	1	100017	9	2	6	1	2
1	1	100018	40	1	108	2	2
1	1	100019	54	2	120	1	2
1	1	100020	32	2	57	1	2
1	1	100021	43	1	276	2	3
1	1	100022	47	1	108	1	2
1	1	100023	41	1	60	1	2
1	1	100024	59	1	120	1	1
1	1	100025	62	1	120	1	2
1	2	100026	51	2	24	1	2
1	2	100027	64	1	120	1	2
1	2	100028	74	1	36	1	2
1	2	100029	54	2	300	1	1
1	2	100030	34	2	12	1	2
1	2	100031	33	2	72	1	2
1	2	100032	24	1	36	1	1
1	2	100033	26	1	36	1	2
1	2	100034	52	1	120	1	3
1	2	100035	38	2	156	1	2
1	2	100036	59	1		1	3
1	2	100037	45	1	310	1	3
1	2	100038	57	1	60	1	2
1	2	100039	31	1	12	1	2
1	2	100040	35	1	72	1	2
1	2	100041	67	2	300	2	2
1	2	100042	51	2	60	1	2
1	2	100043	38	2	144	1	2
1	2	100044	68	2	60	1	2
1	3	100045	42	2	36	1	2
1	3	100046	18	2	60	1	2
1	3	100047	42	1	72	1	2
1	3	100048	51	2	60	1	2
1	3	100049	44	2	36	1	3

1	3	100050	29	1	24	1	3
1	3	100051	61	1	72	1	2
1	3	100052	30	2	18	1	2
1	3	100053	56	1	36	2	2
1	3	100054	63	2	0	1	2
1	3	100055	26	2	120	1	3
1	3	100056	60	2	24	1	1
1	3	100057	27	1	6	1	2
1	3	100058	41	1	24	1	
1	3	100059	31	1	36	1	2
1	3	100060	54	1	36	1	2
1	3	100061	72	1	240	3	3
1	3	100062	67	2	72	2	2
1	4	100063	37	1	120	1	3
1	4	100064	58	1	336	2	3
1	4	100065	31	1	0	1	3
1	4	100066	58	1	72	1	2
1	4	100067	40	1	24	1	2
1	4	100068	37	1	24	1	3
1	4	100069	42	2	48	1	2
1	4	100070	52	2	156	2	3
1	4	100071	58	1	48	1	2
1	4	100072	61	2	17	1	2
1	4	100073	68	2	114	1	2
1	4	100074	71	1	48	1	2
1	4	100075	37	2	12	1	2
1	4	100076	35	1	60	1	3
1	4	100077	60	2		1	2
1	4	100078	34	2	12	1	2
1	4	100079	48	1	36	1	3
1	5	100080	40	1	48	1	2
1	5	100081	49	1	120	1	2
1	5	100082	57	1	132	1	2
1	5	100083	33	1	180	1	3
1	5	100084	30	2	72	1	2
1	5	100085	54	1	72	1	3
1	5	100086	72	2	84	1	2
1	5	100087	57	2	36	1	2
1	5	100088	56	2	143	1	2
1	5	100089	80	1	84	2	2
1	5	100090	45	1	72	1	2
1	5	100091	40	1		1	1
1	5	100092	56	1	84	1	2
1	5	100093	62	1	72	1	2
1	5	100094	31	2	10	1	2
1	5	100095	44	1	240	3	3
1	6	100096	40	1	24	1	2
1	6	100097	31	1	96	1	3
1	6	100098	44	1	60	1	2
1	6	100099	35	2	24	1	3
1	6	100100	51	1	96	1	3
1	6	100101	40	1	120	2	3
1	6	100102	33	1	240	2	3
1	6	100103	23	1	48	1	3
1	6	100104	46	1	36	1	3
1	6	100105	39	1	12	1	2
1	6	100106	65	1	36	2	1
1	6	100107	46	2	120	1	2
1	6	100108	30	1	21	1	3

1	6	100109	57	1	12	1	2
1	6	100110	49	1	48	1	2
1	7	100111	46	2	12	1	2
1	7	100112	49	1	30	2	3
1	7	100113	29	2	24	1	2
1	7	100114	50	2	120	1	3
1	7	100115	32	2	24	1	2
1	7	100116	41	1	60	1	1
1	7	100117	24	2	12	1	3
1	7	100118	31	2	72	1	2
1	7	100119	30	2	24	1	2
1	7	100120	21	1	36	1	3
1	7	100121	42	1	72	1	2
1	7	100122	79	1	x	x	
1	7	100123	49	1	240	1	2
1	7	100124	30	1	96	2	3
1	7	100125	78	2	420	1	2
1	8	100126	30	2	48	1	1
1	8	100127	39	2	36	1	2
1	8	100128	63	2	360	1	3
1	8	100129	27	2	72	1	3
1	8	100130	51	1	36	1	2
1	8	100131	62	2	60	1	2
1	8	100132	38	2	16	1	2
1	8	100133	66	1	180	1	1
1	8	100134	54	1	144	3	2
1	8	100135	47	2	4	1	2
1	8	100136	34	1	8	1	2
1	8	100137	56	1	240	1	2
1	8	100138	73	1	96	2	1
1	8	100139	28	1	24	1	2
1	8	100140	41	2	24	1	1
1	9	100141	67	2	120	1	3
1	9	100142	27	1		1	2
1	9	100143	22	2	84	1	2
1	9	100144	36	1	12	1	2
1	9	100145	37	2	240	1	1
1	9	100146	21	2	36	1	2
1	9	100147	44	1	12	1	2
1	9	100148	43	2	36	1	2
1	9	100149	28	1	144	2	3
1	9	100150	23	1	48	1	2
1	9	100151	26	1	24	2	3
1	9	100152	34	2	120	2	3
1	9	100153	36	1	36	1	3
1	9	100154	74	2	120	1	1
1	10	100155	16	1	6	1	1
1	10	100156	48	2	24	1	2
1	10	100157	35	1	60	1	2
1	10	100158	77	2	12	1	2
1	10	100159	60	2	12	1	2
1	10	100160	39	1	96	1	3
1	10	100161	35	2	36	1	2
1	10	100162	59	1	48	1	2
1	10	100163	37	1	60	1	2
1	10	100164	30	1	18	1	2
1	10	100165	49	1	408	2	3
1	10	100166	68	2	24	1	3
1	10	100167	64	2	24	1	3
1	10	100168	48	2	72	1	2
1	11	100169	33	1	24	1	3

1	11	100170	41	1	84	1	2
1	11	100171	24	2	120	1	2
1	11	100172	38	1	60	2	3
1	11	100173	49	2	60	1	2
1	11	100174	52	1	60	1	2
1	11	100175	42	1	42	1	2
1	11	100176	29	1	12	1	2
1	11	100177	75	2	36	2	3
1	11	100178	15	1	48	1	2
1	11	100179	33	1	60	1	2
1	11	100180	63	2	24	1	1
1	11	100181	61	2	12	1	2
1	12	100182	28	2	48	1	2
1	12	100183	47	2	48	1	3
1	12	100184	38	2	26	1	1
1	12	100185	36	2	60	1	2
1	12	100186	64	1	36	1	2
1	12	100187	33	2	120	1	2
1	12	100188	82	1	360	2	2
1	12	100189	42	2	36	1	3
1	12	100190	37	1	72	1	2
1	12	100191	61	2	120	1	2
1	12	100192	56	1	60	1	2
1	12	100193	52	2	132	1	2
1	12	100194	71	1	480	2	3
1	13	100195	39	2	156	2	2
1	13	100196	35	1	16	1	2
1	13	100197	55	1	156	1	2
1	13	100198	63	1	48	2	3
1	13	100199	42	1	36	1	3
1	13	100200	31	2	48	1	2
1	13	100201	58	2	300	1	3
1	13	100202	67	1	456	2	2
1	13	100203	20	1	12	1	2
1	13	100204	50	1	120	1	3
1	13	100205	34	2	120	2	3
1	13	100206	41	1	60	1	2
1	13	100207	57	1	132	1	3
1	14	100208	28	1	48	1	3
1	14	100209	45	1	120	1	2
1	14	100210	26	2	3	1	3
1	14	100211	30	2	96	1	2
1	14	100212	42	2	96	1	2
1	14	100213	53	2	60	1	1
1	14	100214	68	2	20	1	1
1	14	100215	65	2	24	1	2
1	14	100216	48	1	120	1	3
1	14	100217	50	1	144	2	2
1	14	100218	35	1	36	2	3
1	14	100219	39	1	120	3	3
1	15	100220	76	1	72	1	2
1	15	100221	64	2	24	1	3
1	15	100222	77	1	240	2	3
1	15	100223	31	1	36	1	3
1	15	100224	66	2	600	1	2
1	15	100225	27	2	96	1	2
1	15	100226	51	2	432	1	2
1	15	100227	75	2	240	1	2
1	15	100228	43	1	120	1	2
1	15	100229	25	2	12	1	3
1	15	100230	46	1	60	1	3
1	16	100231	44	2	240	1	2
1	16	100232	27	2	180	1	2
1	16	100233	30	2	180	1	1

1	16	100234	29	2	48	1	1
1	16	100235	26	2	120	1	3
1	16	100236	45	1	18	1	2
1	16	100237	56	1	12	1	2
1	16	100238	59	1	144	2	2
1	16	100239	54	1	372	2	3
1	16	100240	31	1	48	1	2
1	16	100241	35	1	36	2	3
2	17	100242	44	1	60	1	2
2	17	100243	28	2	84	1	3
2	17	100244	29	1	12	1	2
2	17	100245	19	1	60	1	3
2	17	100246	22	1	12 x	x	
2	17	100247	45	1	12	1	1
2	17	100248	48	1	120	1	3
2	17	100249	31	1	12	1	3
2	17	100250	62	1	144	1	2
2	18	100251	29	1	30	1	2
2	18	100252	41	2	60	1	2
2	18	100253	28	2	36	1	1
2	18	100254	32	1	60	1	2
2	18	100255	70	2	36	1	2
2	18	100256	49	2	120	1	2
2	18	100257	50	2	240	2	2
2	18	100258	25	2	120	1	2
2	18	100259	33	2	192	3	3
2	19	100260	47	2	60	1	2
2	19	100261	62	2	40	1	2
2	19	100262	29	2	12	1	1
2	19	100263	22	1	84	1	2
2	19	100264	63	1	6	1	1
2	19	100265	55	2	120	1	2
2	19	100266	31	1	144	1	2
2	19	100267	33	2	18	1	2
2	20	100268	37	1	120	1	3
2	20	100269	25	2	12	1	3
2	20	100270	40	1	360	1	3
2	20	100271	57	2	72	1	2
2	20	100272	49	2	252	2	3
2	20	100273	73	1	24	2	2
2	20	100274	47	1	6	1	2
2	20	100275	44	1	12	1	2
2	21	100276	32	2	60	1	3
2	21	100277	57	1	72	1	2
2	21	100278	69	2	84	1	2
2	21	100279	45	1	225	2	3
2	21	100280	49	1	180	1	3
2	21	100281	29	1	36	1	2
2	21	100282	29	2	36	1	2
2	21	100283	65	1	480	2	3
2	22	100284	44	2	72	1	3
2	22	100285	50	1	84	1	3
2	22	100286	71	2	3	1	3
2	22	100287	64	1	48	1	2
2	22	100288	42	1	12	1	2
2	22	100289	67	1	120	1	2
2	22	100290	25	1	48	1	2
2	22	100291	60	1	240	1	2
2	23	100292	75	2	324	1	3
2	23	100293	52	2	120	1	3
2	23	100294	56	1	120	1	2

2	23	100295	29	1	96	1	3
2	23	100296	53	2	120	2	3
2	23	100297	59	2	180	1	3
2	23	100298	61	2	360	2	3
2	23	100299	70	2	600	3	3
2	24	100300	24	2	18	1	2
2	24	100301	26	2	7	1	2
2	24	100302	69	1	300	1	2
2	24	100303	38	1	12	1	1
2	24	100304	54	1	72	1	2
2	24	100305	50	2	48	2	2
2	24	100306	69	1	18	2	3
2	24	100307	68	2	48	2	3
2	25	100308	37	2	96	1	1
2	25	100309	50	2	180	2	2
2	25	100310	54	1	12	1	2
2	25	100311	38	1	180	2	3
2	25	100312	61	1	84	1	1
2	25	100313	23	2	24	1	2
2	25	100314	25	1	24	1	3
2	25	100315	21	1	24	1	2
2	26	100316	30	1	72	1	1
2	26	100317	38	1	36	1	3
2	26	100318	33	1	10	1	3
2	26	100319	48	2	36	1	1
2	26	100320	34	1	420	2	2
2	26	100321	74	2	24	1	2
2	26	100322	43	1	144	3	3
2	26	100323	55	1	10	1	1
2	27	100324	36	1	72	1	2
2	27	100325	47	2	108	2	3
2	27	100326	29	1	192	3	2
2	27	100327	58	2	72	1	2
2	27	100328	48	1	8	1	2
2	27	100329	47	2	48	1	3
2	27	100330	46	1	16	1	2
2	27	100331	62	1	240	3	3
2	28	100332	55	2	120	1	2
2	28	100333	23	2	48	1	1
2	28	100334	48	2	36	1	2
2	28	100335	36	1	18	1	2
2	28	100336	28	1	120	1	2
2	28	100337	30	2	24	1	2
2	28	100338	74	1	120	3	3
2	29	100339	59	2	108	2	2
2	29	100340	48	1	216	2	3
2	29	100341	46	1	372	3	3
2	29	100342	46	2	144	1	2
2	29	100343	61	1	180	2	3
2	29	100344	36	1	180	1	3
2	30	100345	39	2	27	1	3
2	30	100346	68	2	12	1	1
2	30	100347	46	1	36	1	3
2	30	100348	73	1	6	2	2
2	30	100349	55	1	480	1	2
2	30	100350	71	1	4	1	2
2	31	100351	76	2	78	1	2
2	31	100352	62	2	24	1	2
2	31	100353	48	2	48	2	2
2	31	100354	33	1	12	1	2
2	31	100355	49	1	120	2	2
2	31	100356	83	2	8	1	2
2	32	100357	49	2	60	1	2
2	32	100358	41	2	108	2	3

2	32	100359	53	1	60	1	3
2	32	100360	13	1	13	1	2
2	32	100361	62	2	36	1	2
2	32	100362	44	1	6	1	1
2	33	100363	30	1	60	2	3
2	33	100364	63	2	396	3	3
2	33	100365	47	2	120	1	2
2	33	100366	29	2	12	1	2
2	33	100367	37	2	26	1	2
2	33	100368	38	1	12	1	1
2	34	100369	51	2	264	1	2
2	34	100370	42	1	24	1	2
2	34	100371	28	1	36	1	1
2	34	100372	38	1	12	1	3
2	34	100373	62	1	24	1	2
2	34	100374	52	2	120	2	3
2	35	100375	52	1	15	1	2
2	35	100376	58	2	24	1	3
2	35	100377	49	1	12	1	2
2	35	100378	71	1	12 x	x	
2	35	100379	40	1	240	2	3
2	35	100380	26	1	96	1	2
2	36	100381	67	2	60	3	3
2	36	100382	33	2	36	1	1
2	36	100383	45	1	36	1	3
2	36	100384	38	1	192	3	3
2	36	100385	57	1	18	1	2
2	37	100386	38	1	72	1	3
2	37	100387	37	1	4	1	1
2	37	100388	60	2	360	3	2
2	37	100389	47	1	36	1	2
2	37	100390	61	2	60	2	2
2	38	100391	55	1	24	1	2
2	38	100392	38	1	24		
2	38	100393	67	1	120	1	2
2	38	100394	43	2	48	1	3
2	38	100395	57	1	18	1	2
2	39	100396	34	2	29	1	1
2	39	100397	58	2	48	1	2
2	39	100398	38	1	36	1	1
2	39	100399	75	2	240	1	2
2	40	100400	27	1	24	1	3
2	40	100401	33	2	60	1	2
2	40	100402	55	2		1	1
2	40	100403	60	2	72	1	1
2	41	100404	72	2	60	2	2
2	41	100405	32	1	48	1	3
2	41	100406	31	1	120	1	3
2	41	100407	40	2	120	3	3
2	42	100408	42	1	13	1	2
2	42	100409	46	2	48	1	2
2	42	100410	42	1	9	1	2
2	42	100411	44	1	60	1	2
2	43	100412	52	2	180	1	2
2	43	100413	67	2	120	1	2
2	43	100414	29	2	96	1	2
2	43	100415	35	2	360	2	2
2	44	100416	65	1	36	1	3
2	44	100417	36	1	12	1	2
2	44	100418	47	1	24	1	1
2	44	100419	52	1	180	1	3
2	45	100420	46	1	120	1	3
2	45	100421	70	1	36	1	1
2	45	100422	33	2	48	1	2

2	45	100423	48	1	120	1	2
2	46	100424	22	1	18	1	2
2	46	100425	58	2	72	1	2
2	46	100426	44	1	36	1	3
2	47	100427	23	2	60	1	2
2	47	100428	35	2	180	1	2
2	47	100429	17	1	12	1	1
2	48	100430	58	2	240	1	2
2	48	100431	81	2	36		2
2	48	100432	52	2	24	1	2
2	49	100433	61	2	36	1	2
2	49	100434	73	2	120	2	
2	49	100435	71	1		1	1
2	50	100436	40	1	192	1	3
2	50	100437	48	1	10	1	1
2	50	100438	73	1	20	1	1
2	51	100439	35	1	36	3	3
2	51	100440	47	1		1	2
2	51	100441	58	2		1	1
2	52	100442	41	1	24	1	3
2	52	100443	47	1	12	1	2
2	52	100444	78	2	48	2	2
2	53	100445	38	1	6	1	1
2	53	100446	30	1	120	1	2
2	53	100447	44	2	36	1	2
2	54	100448	62	1	60	2	3
2	54	100449	42	2	312	1	1
2	54	100450	31	2	36	1	2
2	55	100451	61	2		1	2
2	55	100452	67	2	17	1	2
2	56	100453	80	1		1	3
2	56	100454	42	1	6	1	2
2	57	100455	56	1	24	1	2
2	57	100456	68	2	120	3	3
2	58	100457	59	2	6	2	3
2	58	100458	44	1	12	1	2
2	59	100459	73	1	600	1	1
2	60	100460	45	1	72	2	2
2	61	100461	72	2	420	1	2
2	62	100462	61	1	96	1	2
2	63	100463	21	1	6	1	1

maximum transverse diameter (mm)	surgical approach	methods	surgeon identity code	operative time (min)	blood loss (ml)	intraoperative complication (0=No,1=Yes)	Nasogastric tube was removed (POD)	water intake was started (POD)	food intake was started (POD)
	laparoscop	Heller-Dor	OMU	120	0	0			
45	laparoscop	Heller-Dor	AOK	140	0	0			
66	laparoscop	Heller-Dor	KAW	145	0	0	0	1	2
30	laparoscop	Heller-Dor	SAS	154	0	0	0	1	2
60	laparoscop	Heller-Dor	WAT	160	10	0	0	1	2
65	laparoscop	Heller-Dor	MAT	160	0	0	0	1	2
45	laparoscop	Heller-Dor	MIT	174	5	0	0	1	2
45	laparoscop	Heller-Dor	YUD	174	0	0	0	1	2
42	laparoscop	Heller-Dor	NIS	174	0	0	0	1	2
45	laparoscop	Heller-Dor	YAN	180	0	1			
57	laparoscopi	Heller-Dor	ISH	190	1300	1	2	2	2
25	laparoscop	Heller-Dor	HAN	190	0	0			
40	laparoscop	Heller-Dor	TAK	200	0	0	0	1	2
55	laparoscop	Heller-Dor	TSU	200	170	1			
46	laparoscop	Heller-Dor	YET	210	0	0	1	1	2
39	laparoscop	Heller-Dor	ETO	211	0	0	0	1	2
45	laparoscop	Heller-Dor	YOS	215	0	0	0	1	2
50	laparoscop	Heller-Ant	NAK	223	200	1	0	1	2
50	laparoscop	Heller-Dor	YAM	229	0	0	0	1	2
	laparoscop	Heller-Dor	KAS	235	0	0			
76	laparoscop	Heller-Dor	YAJ	260	0	1	0		3
55	laparoscop	Heller-Dor	TAN	263	50	0	0	1	2
50	laparoscop	Heller-Dor	MAS	281	0	0	0	1	2
30	laparoscop	Heller-Dor	AKI	296	50	0	0	1	2
45	laparoscop	Heller-Dor	HOS	447	621	1	0	1	5
48	laparoscop	Heller-Dor	OMU	110	0	0			
52	laparoscop	Heller-Dor	AOK	150	0	0			
40	laparoscop	Heller-Dor	WAT	165	20	0	0	1	2
30	laparoscop	Heller-Dor	SAS	168	0	0	0	1	2
50	laparoscop	Heller-Dor	TSU	170	0	0			
55	laparoscop	Heller-Dor	KAW	170	0	0	0	1	2
30	laparoscop	Heller-Dor	ISH	180	0	0			
58	laparoscop	Heller-Dor	YUD	187	0	0	0	1	2
60	laparoscop	Heller-Dor	YAM	191	50	0	0	1	2
52.3	laparoscop	Heller-Dor	YAN	200	0	0			
	laparoscop	Heller-Dor	KAS	205	0	0			
70	laparoscop	Heller-Dor	TAN	206	120	1	0	1	2
52	laparoscop	Heller-Dor	MAT	215	50	1	0	1	2
40	laparoscop	Heller-Dor	AKI	220	0	0	0	1	2
40	laparoscop	Heller-Dor	NAK	227	0	0	0	1	2
46	laparoscop	Heller-Dor	ETO	230	0	1	0	1	2
50	laparoscop	Heller-Dor	HAN	240	0	1	0	4	4
48	laparoscop	Heller-Dor	NIS	258	28	1	0	3	6
45	laparoscop	Heller-Dor	HOS	304	0	1	0	1	2
49	laparoscop	Heller-Dor	KAW	120	20	0	0	1	2
40	laparoscop	Heller-Dor	OMU	120	0	0	0	1	2
47	laparoscop	Heller-Dor	YAN	140	0	0			
40	laparoscop	Heller-Dor	TAN	157	10	0	0	1	2
63	laparoscop	Heller-Dor	AOK	160	0	0			

70	laparoscopy	Heller-Dor	SAS	160	0	0	0	1	2
40	laparoscopy	Heller-Dor	ISH	165	0	0	0	1	2
55	laparoscopy	Heller-Dor	WAT	165	10	0	0	1	2
55	laparoscopy	Heller-Dor	KAS	170	0	0			
44	laparoscopy	Heller-Dor	NIS	173	20	1	0	1	2
60	laparoscopy	Heller-Dor	MAT	190	20	1	0	1	2
30	laparoscopy	Heller-Dor	YUD	205	0	1	0	1	2
50	laparoscopy	Heller-Dor	TSU	215	0	0	0	1	2
	laparoscopy	Heller-Dor	YAM	252	50	0	0	1	2
45	laparoscopy	Heller-Dor	HOS	273	0	0	0	1	2
50	laparoscopy	Heller-Dor	NAK	275	0	1	0	2	3
70	laparoscopy	Heller-Dor	AKI	301	150	0	0	1	2
37	laparoscopy	Heller-Dor	ETO	317	0	1	0	1	2
70	laparoscopy	Heller-Dor	OMU	120	0	0	0	1	2
67	laparoscopy	Heller-Dor	YAN	140	0	0	0	1	2
80	laparoscopy	Heller-Dor	KAS	150	0	1			
35	laparoscopy	Heller-Dor	SAS	155	0	0	0	1	2
49	laparoscopy	Heller-Dor	MAT	155	100	1	0	1	2
70	laparoscopy	Heller-Dor	WAT	186	0	0	0	1	2
38	laparoscopy	Heller-Dor	TSU	190	0	0	0	1	2
60	laparoscopy	Heller-Dor	ISH	205	0	0	0	2	3
47	laparoscopy	Heller-Dor	KAW	210	0	0	0	1	2
55	laparoscopy	Heller-Dor	NAK	216	0	0	0	1	2
40	laparoscopy	Heller-Dor	YUD	217	0	0	0	1	2
50	laparoscopy	Heller-Dor	HOS	219	0		0	1	2
45	laparoscopy	Heller-Dor	AOK	220	0	1			
70	laparoscopy	Heller-Dor	TAN	228	90	1	0	1	4
38	laparoscopy	Heller-Dor	AKI	229	0	0	0	1	2
35	laparoscopy	Heller-Dor	YAM	230	0	1	0	1	2
70	laparoscopy	Heller-Dor	NIS	248	20	0	0	1	2
	laparoscopy	Heller-Dor	SAS	124	0	0	0	1	2
40	laparoscopy	Heller-Dor	YAN	173	50	0	0	1	2
46	laparoscopy	Heller-Dor	MAT	175	50	0	0	1	2
65	laparoscopy	Heller-Dor	NIS	175	0	0	0	1	2
58	laparoscopy	Heller-Dor	ISH	180	0	0	0	1	2
65	laparoscopy	Heller-Dor	OMU	180	100	0	1	2	3
40	laparoscopy	Heller-Dor	TAN	184	30	0	0	1	2
45	laparoscopy	Heller-Dor	KAW	190	0	1	0	1	2
46	laparoscopy	Heller-Dor	WAT	193	20	0	0	1	2
55	laparoscopy	Heller-Dor	YAM	205	150	0	0	1	2
47	laparoscopy	Heller-Dor	YUD	218	0	0	0	1	2
28	laparoscopy	Heller-Dor	AKI	220	0	0	0	1	2
46.6	laparoscopy	Heller-Dor	TSU	230	0	0	0	1	2
	laparoscopy	Heller-Dor	KAS	235	0	1			
52	laparoscopy	Heller-Dor	NAK	255	0	0	0	1	2
	laparoscopy	Heller-Dor	HOS	281	50		0	1	2
50	laparoscopy	Heller-Dor	OMU	120	200	1			
60	laparoscopy	Heller-Dor	SAS	123	0	0	0	1	2
43	laparoscopy	Heller-Dor	KAS	145	0	0			
60	laparoscopy	Heller-Dor	MAT	155	0	0	0	1	2
80	laparoscopy	Heller-Dor	YAN	160	110	0	0	1	5
70	laparoscopy	Heller-Dor	KAW	172	0	0	0	1	2
100	laparoscopy	Heller-Dor	ISH	180	0	0	0	1	2
61	laparoscopy	Heller-Dor	NIS	182	0	0	0	1	2
64	laparoscopy	Heller-Dor	AKI	195	0	0	0	1	2
46	laparoscopy	Heller-Dor	TSU	200	150	0	0	1	2
23	laparoscopy	Heller-Dor	YAM	211	0	0	0	1	2
40	laparoscopy	Heller-Dor	YUD	221	0	0	0	1	2
85	laparoscopy	Heller-Dor	NAK	226	0	1	0	1	2

35	laparoscopy	Heller-Dor	TAN	241	90	1	0	1	2
50	laparoscopy	Heller-Dor	HOS	243	0		0	1	2
	laparoscopy	Heller-Dor	SAS	133	0	0	0	1	2
61	laparoscopy	Heller-Dor	OMU	135	0	0			
45	laparoscopy	Heller-Dor	KAS	155	0	0			
67	laparoscopy	Heller-Dor	AKI	160	0	0	0	1	2
41	laparoscopy	Heller-Dor	TSU	165	0	0	0	1	2
34	laparoscopy	Heller-Dor	KAW	170	0	0	0	1	2
70	laparoscopy	Heller-Dor	ISH	175	0	0	0	1	2
38	laparoscopy	Heller-Dor	TAN	182	20	0	0	1	2
48	laparoscopy	Heller-Dor	NIS	187	0	1	0	1	2
61	laparoscopy	Heller-Dor	YAN	190	20	1	0	1	2
57	laparoscopy	Heller-Dor	MAT	200	0	0	0	1	2
	laparoscopy	Heller-Dor	YAM	216	0	0	0	1	2
42	laparoscopy	Heller-Dor	HOS	241	0		0	1	2
72	laparoscopy	Heller-Dor	NAK	265	0	0	0	1	2
	laparoscopy	Heller-Dor	YUD	274	150	1	0	1	2
32	laparoscopy	Heller-Dor	SAS	112	0	0	0	1	2
38	laparoscopy	Heller-Dor	KAW	125	0	1	0	7	9
65	laparoscopy	Heller-Dor	KAS	145	0	0			
61	laparoscopy	Heller-Dor	OMU	150	0	1	1	3	4
46	laparoscopy	Heller-Dor	MAT	160	0	0	0	1	2
37.5	laparoscopy	Heller-Dor	ISH	170	0	1	0	1	2
36	laparoscopy	Heller-Dor	TSU	170	0	0	0	1	2
32	laparoscopy	Heller-Dor	AKI	182	0	0	0	1	2
55	laparoscopy	Heller-Dor	NAK	187	0	0	0	1	2
36	laparoscopy	Heller-Dor	NIS	187	10	0	0	1	2
42	laparoscopy	Heller-Dor	YAN	197	20	1	0	1	2
52	laparoscopy	Heller-Dor	TAN	199	20	0	0	1	2
13	laparoscopy	Heller-Dor	YAM	209	0	0	0	1	2
55	laparoscopy	Heller-Dor	HOS	267	0	0	0	1	2
30	laparoscopy	Heller-Dor	YUD		0	0	0	1	2
65	laparoscopy	Heller-Dor	SAS	115	0	0	0	1	2
48	laparoscopy	Heller-Dor	NIS	150	5	0	0	1	2
48	laparoscopy	Heller-Dor	KAW	155	0	0	0	1	2
47	laparoscopy	Heller-Dor	YAN	155	0	0	0	1	2
33	laparoscopy	Heller-Dor	AKI	158	0	0	0	1	2
52	laparoscopy	Heller-Dor	TAN	162	10	0	0	1	2
58	laparoscopy	Heller-Dor	TSU	165	0	0	0	1	2
50	laparoscopy	Heller-Dor	KAS	175	0	0	0	1	2
70	laparoscopy	Heller-Dor	OMU	180	0	0	0	1	2
36	laparoscopy	Heller-Dor	MAT	185	0	0	0	1	2
62	laparoscopy	Heller-Dor	ISH	189	0	0	0	1	2
64	laparoscopy	Heller-Dor	NAK	195	0	0	0	1	2
62	laparoscopy	Heller-Dor	YAM	224	0	0	0	1	2
25	laparoscopy	Heller-Dor	HOS	241	0	0	0	1	2
28	laparoscopy	Heller-Dor	TAN	139	0	0	0	1	2
56	laparoscopy	Heller-Dor	SAS	140	0	0	0	1	2
44	laparoscopy	Heller-Dor	OMU	150	0	0	0	1	2
38	laparoscopy	Heller-Dor	NIS	156	5	0	0	1	2
54	laparoscopy	Heller-Dor	NAK	165	0	0	0	1	2
67	laparoscopy	Heller-Dor	MAT	165	0	0	0	1	2
42	laparoscopy	Heller-Dor	TSU	170	0	0	0	1	2
42	laparoscopy	Heller-Dor	KAW	190	0	1	0	2	3
50	laparoscopy	Heller-Dor	ISH	190	0	0	0	1	2
37	laparoscopy	Heller-Dor	AKI	193	0	0	0	1	2
67	laparoscopy	Heller-Dor	YAM	214	0	0	0	1	2
	laparoscopy	Heller-Dor	KAS	215	0	0			
60	laparoscopy	Heller-Dor	HOS	228	0	0	0	1	2
50	laparoscopy	Heller-Dor	YAN	262	0	0	0	1	2
70	laparoscopy	Heller-Dor	TSU	120	0	0	0	1	2

45	laparoscopy	Heller-Dor	TAN	138	0	0	0	1	2
42	laparoscopy	Heller-Dor	YAN	140	0	0	0	1	2
75	laparoscopy	Heller-Dor	KAS	155	0	0			
52	laparoscopy	Heller-Dor	SAS	164	0	1	0	1	2
47	laparoscopy	Heller-Dor	YAM	174	0	0	0	1	2
57	laparoscopy	Heller-Dor	OMU	185	0	0	0	1	2
62	laparoscopy	Heller-Dor	NIS	198	5	0	0	1	2
65	laparoscopy	Heller-Dor	MAT	200	100	1	0	1	0
43	laparoscopy	Heller-Dor	KAW	210	0	0	0	1	2
50	laparoscopy	Heller-Dor	ISH	215	0	0	0	1	2
25	laparoscopy	Heller-Dor	NAK	235	0	1	0	1	2
40	laparoscopy	Heller-Dor	HOS	285	0	1	0	1	2
59	laparoscopy	Heller-Dor	OMU	110	0	0	0	1	1
	laparoscopy	Heller-Dor	SAS	110	0	0	0	1	2
27	laparoscopy	Heller-Dor	TSU	130	0	0	0	1	2
45	laparoscopy	Heller-Dor	YAN	150	0	0	0	1	7
37	laparoscopy	Heller-Dor	NIS	154	5		0	1	2
42	laparoscopy	Heller-Dor	KAW	172	0	1	0	1	2
55	laparoscopy	Heller-Dor	KAS	180	100	1	1	6	9
68	laparoscopy	Heller-Dor	TAN	194	0	0	0	1	2
51	laparoscopy	Heller-Dor	YAM	200	0	0	0	1	2
41	laparoscopy	Heller-Dor	MAT	210	0	1	0	1	2
36	laparoscopy	Heller-Dor	NAK	220	0	0	0	1	2
46	laparoscopy	Heller-Dor	ISH	230	0	0	0	1	2
60	laparoscopy	Heller-Dor	HOS	232	0	0	0	1	2
50	laparoscopy	Heller-Dor	KAW	110	0	0	0	1	2
55	laparoscopy	Heller-Dor	TSU	135	0	0	0	1	2
56	laparoscopy	Heller-Dor	OMU	140	0	0	0	6	7
66	laparoscopy	Heller-Dor	SAS	155	0	0	0	1	2
60	laparoscopy	Heller-Dor	TAN	161	0	0	0	1	2
40	laparoscopy	Heller-Dor	KAS	170	0	0			
65	laparoscopy	Heller-Dor	NAK	175	0	0	0	1	2
52	laparoscopy	Heller-Dor	NIS	175	15	1	0	1	2
57	laparoscopy	Heller-Dor	YAN	180	0	0	0	1	2
65	laparoscopy	Heller-Dor	HOS	180	0	0	0	1	2
63	laparoscopy	Heller-Dor	YAM	200	0	0	0	1	2
50	laparoscopy	Heller-Dor	MAT	210	50	0	0	1	2
90	laparoscopy	Heller-Dor	ISH	240	0	1	0	1	2
73	laparoscopy	Heller-Dor	OMU	130	0	0	0	1	2
55	laparoscopy	Heller-Dor	SAS	132	0	0	0	1	2
67	laparoscopy	Heller-Dor	TSU	155	0	0	0	1	2
50	laparoscopy	Heller-Dor	ISH	160	0	0	0	1	2
40	laparoscopy	Heller-Dor	MAT	170	0	0	0	1	2
25	laparoscopy	Heller-Dor	NIS	172	0	0	0	1	2
10	laparoscopy	Heller-Dor	HOS	180	0	0	0	1	2
37	laparoscopy	Heller-Dor	KAS	180	100	0			
60	laparoscopy	Heller-Dor	TAN	182	20	0	0	1	2
48	laparoscopy	Heller-Dor	KAW	210	0	0	0	1	2
62	laparoscopy	Heller-Dor	YAM	215	0	0	0	1	2
72	laparoscopy	Heller-Ante	YAN	266	90	0	0	1	2
50	laparoscopy	Heller-Dor	KAS	130	0	0	2	3	4
60	laparoscopy	Heller-Dor	SAS	133	0	0	0	1	2
69	laparoscopy	Heller-Dor	OMU	138	0	0	0	1	3
60	laparoscopy	Heller-Dor	TAN	160	0	0	0	1	2
61	laparoscopy	Heller-Dor	YAM	160	0	1	0	1	2
45	laparoscopy	Heller-Dor	YAN	164	15	0	0	1	2
50	laparoscopy	Heller-Dor	TSU	180	0	0	0	1	2
54	laparoscopy	Heller-Dor	ISH	185	0	0	0	1	2
55	laparoscopy	Heller-Dor	NIS	185	10	0	0	1	2
68	laparoscopy	Heller-Dor	MAT	195	30	0	0	1	4
67	laparoscopy	Heller-Dor	KAW	195	0	0	0	1	2
35.6	laparoscopy	Heller-Dor	OMU	135	0	0	0	1	3
35	laparoscopy	Heller-Dor	SAS	144	0	0	0	1	2
34	laparoscopy	Heller-Dor	YAM	160	0	0	0	1	2

24	laparoscopy	Heller-Dor	ISH	160	0	0	0	1	8
82	laparoscopy	Heller-Dor	KAS	160	0	0			
48	laparoscopy	Heller-Dor	YAN	167	60	0	0	1	2
48	laparoscopy	Heller-Dor	TAN	172	0	0	0	1	2
45	laparoscopy	Heller-Dor	MAT	177	0	0	0	1	2
60	laparoscopy	Heller-Dor	TSU	190	0	0	0	1	2
50	laparoscopy	Heller-Dor	KAW	190	0	0	0	1	2
84	laparoscopy	Heller-Dor	NIS	215	0	0	0	1	2
55	laparoscopy	Heller-Dor	KAW	120	0	0	0	1	2
65	laparoscopy	Heller-Dor	TSU	120	0	0	0	1	2
42	laparoscopy	Heller-Dor	MAT	135	0	0	0	1	2
73	laparoscopy	Heller-Dor	YAN	138	0	1	0	1	2
	laparoscopy	Heller-Dor	YAM	165	0	0	0	1	2
29	laparoscopy	Heller-Dor	ISH	171	0	0	0	1	2
83	laparoscopy	Heller-Dor	OMU	190	50	0			
60	laparoscopy	Heller-Dor	KAS	205	0	1			
55	laparoscopy	Heller-Dor	TAN	219	50	0	0	1	2
38	laparoscopy	Heller-Dor	OMU	125	0	0	0	1	2
45	laparoscopy	Heller-Dor	MAT	130	0	0	0	1	2
33	laparoscopy	Heller-Dor	TSU	135	50	0	0	1	2
35	laparoscopy	Heller-Dor	KAS	140	0	0			
46	laparoscopy	Heller-Dor	TAN	143	0	0	0	1	2
50	laparoscopy	Heller-Dor	YAN	147	0	0	0	1	2
52	laparoscopy	Heller-Dor	KAW	160	0	0	0	1	2
40	laparoscopy	Heller-Dor	ISH	195	0	1	0	1	2
82	laparoscopy	Heller-Dor	YAM	300	0	1	0	1	2
39	laparoscopy	Heller-Dor	YAM	143	0	0	0	1	2
55	laparoscopy	Heller-Dor	MAT	150	0	1	0	1	2
25	laparoscopy	Heller-Dor	OMU	155	0	0	0	1	2
54	laparoscopy	Heller-Dor	KAS	160	0	0	0	1	2
33.8	laparoscopy	Heller-Dor	TSU	160	50	1	0	3	4
48	laparoscopy	Heller-Dor	ISH	180	0	0	0	1	2
55	laparoscopy	Heller-Dor	YAN	186	0	0	0	1	2
50	laparoscopy	Heller-Dor	KAW	205	0	0	0	1	2
61	laparoscopy	Heller-Dor	TSU	120	0	1	0	1	3
70	laparoscopy	Heller-Dor	MAT	128	0	0	0	1	2
71.2	laparoscopy	Heller-Dor	OMU	135	0	0	0	1	2
55	laparoscopy	Heller-Dor	YAM	140	0	0	0	1	2
82	laparoscopy	Heller-Dor	KAS	145	0	1	0	1	2
55	laparoscopy	Heller-Ante	YAN	162	40	1	0	1	2
59	laparoscopy	Heller-Dor	ISH	165	0	0	0	1	2
57	laparoscopy	Heller-Dor	KAW	190	0	0	0	1	2
62	laparoscopy	Heller-Ante	MAT	140	0	0	0	1	2
45	laparoscopy	Heller-Dor	ISH	145	0	0	0	1	2
35	laparoscopy	Heller-Dor	KAS	150	0	0	0	1	2
70	laparoscopy	Heller-Dor	TSU	150	0	0	0	1	2
68	laparoscopy	Heller-Dor	YAM	151	0	0	0	1	2
45	laparoscopy	Heller-Dor	OMU	165	0	0	0	1	2
51	laparoscopy	Heller-Dor	KAW	205	0	1	0	1	2
92	laparoscopy	Heller-Ante	YAN	278	25	1	0	1	2
67	laparoscopy	Heller-Dor	KAW	100	0	0	0	1	2
65	laparoscopy	Heller-Dor	TSU	100	0	0	0	1	2
70	laparoscopy	Heller-Dor	KAS	120	0	0	0	1	2
42	laparoscopy	Heller-Dor	MAT	130	0	1	0	1	2
35	laparoscopy	Heller-Dor	YAN	132	0	0	0	1	2
44.2	laparoscopy	Heller-Dor	OMU	150	0	0	0	1	2
56	laparoscopy	Heller-Dor	ISH	177	0	0	0	1	2
51	laparoscopy	Heller-Dor	YAM	185	0	0	0	1	2
67	laparoscopy	Heller-Dor	TSU	115	0	0	0	1	2
72	laparoscopy	Heller-Dor	OMU	135	0	1			
52	laparoscopy	Heller-Dor	YAN	151	0	0	0	1	4

60	laparoscopy	Heller-Dor	KAW	160	0	0	0	1	2
82	laparoscopy	Heller-Dor	KAS	190	0	0	0	1	3
70	laparoscopy	Heller-Ante	MAT	195	180	1	0	1	2
71	laparoscopy	Heller-Niss	YAM	213	0	1	0	3	5
70	laparoscopy	Heller-Ante	ISH	253	0	1	0	1	2
35	laparoscopy	Heller-Dor	TSU	115	0	0	0	1	2
38	laparoscopy	Heller-Dor	OMU	120	0	0	0	1	2
47	laparoscopy	Heller-Dor	KAW	120	0	0	0	1	2
30	laparoscopy	Heller-Dor	ISH	146	0	0	0	1	2
37	laparoscopy	Heller-Dor	YAM	159	0	0	0	1	2
50	laparoscopy	Heller-Dor	YAN	161	0	0	0	1	2
70	laparoscopy	Heller-Ante	KAS	167	260	1	0	1	2
60	laparoscopy	Heller-Dor	MAT	180	0	0	0	1	2
30	laparoscopy	Heller-Dor	TSU	115	0	0	0	1	2
55	laparoscopy	Heller-Dor	OMU	130	0	0	0	1	2
44	laparoscopy	Heller-Dor	MAT	135	0	0	0	1	2
88	laparoscopy	Heller-Dor	ISH	142	0	0	0	1	2
21	laparoscopy	Heller-Dor	YAM	144	0	0	0	1	2
50	laparoscopy	Heller-Dor	KAW	155	0	0	0	1	2
72	laparoscopy	Heller-Dor	YAN	174	0	0	0	1	2
35	laparoscopy	Heller-Dor	KAS	178	0	0	0	1	2
30	laparoscopy	Heller-Dor	OMU	130	0	0	0	1	2
65	laparoscopy	Heller-Dor	KAW	130	0	0	0	1	2
62	laparoscopy	Heller-Dor	YAN	135	0	0	0	1	2
33	laparoscopy	Heller-Dor	YAM	143	0	0	0	1	2
52	laparoscopy	Heller-Dor	TSU	145	0	0	0	1	2
40	laparoscopy	Heller-Dor	ISH	210	0	0	0	1	2
72	laparoscopy	Heller-Dor	MAT	225	50	0	0	1	2
22	laparoscopy	Long Heller	KAS	287	0	0	0	1	4
53.3	laparoscopy	Heller-Dor	OMU	110	0	0	0	1	2
60	laparoscopy	Heller-Dor	YAN	115	0	0	0	1	2
55	laparoscopy	Heller-Dor	ISH	132	0	0	0	1	2
53	laparoscopy	Heller-Dor	TSU	135	0	1	0	5	6
45	laparoscopy	Heller-Dor	MAT	145	9	0	0	1	2
60	laparoscopy	Heller-Dor	KAW	170	0	0	0	1	2
56	laparoscopy	Heller-Dor	YAM	180	0	1	0	1	2
70	laparoscopy	Heller-Ante	KAS	226	0	0	0	1	2
40	laparoscopy	Heller-Dor	TSU	95	0	0	0	1	2
30	laparoscopy	Heller-Dor	OMU	110	0	0	0	1	2
40	laparoscopy	Heller-Dor	KAS	141	0	0	0	1	2
53	laparoscopy	Heller-Dor	KAW	150	0	0	0	1	2
39	laparoscopy	Heller-Dor	YAM	156	0	0	0	1	2
42	laparoscopy	Heller-Dor	ISH	173	0	0	0	1	2
125	laparoscopy	Heller-Dor	YAN	279	0	1	0	1	2
46	laparoscopy	Heller-Dor	OMU	104	10	0	0	1	2
70	laparoscopy	Heller-Dor	TSU	155	0	1	0	1	2
69	laparoscopy	Heller-Dor	YAM	160	0	0	0	1	2
53	laparoscopy	Heller-Dor	ISH	178	0	1	0	1	2
105	laparoscopy	Heller-Dor	YAN	191	0	0	0	1	2
98	laparoscopy	Heller-Dor	KAS	205	0	0	0	1	2
	laparoscopy	Heller-Dor	TSU	105	0	0	0	1	2
32	laparoscopy	Heller-Dor	OMU	109	0	0	0	1	2
70	laparoscopy	Heller-Dor	ISH	148	0	0	0	1	2
47	laparoscopy	Heller-Dor	YAM	157	0	0	0	1	2
35	laparoscopy	Heller-Dor	KAS	158	0	0	0	1	2
53	laparoscopy	Heller-Dor	YAN	195	0	0	0	1	2
39	laparoscopy	Heller-Dor	TSU	115	0	0	0	1	2
36	laparoscopy	Heller-Dor	KAS	125	0	0	0	1	2
59	laparoscopy	Heller-Dor	YAM	165	0	0	0	1	2
40	laparoscopy	Heller-Dor	ISH	179	0	0	0	1	2
50	laparoscopy	Heller-Ante	OMU	187	70	1	0	1	2
35	laparoscopy	Heller-Dor	YAN	210	0	0	0	1	2
57	laparoscopy	Heller-Dor	OMU	109	0	0	0	1	2
65	laparoscopy	Heller-Dor	TSU	140	0	0	0	1	2

61	laparoscopy	Heller-Dor	KAS	151	0	0	0	1	2
40	laparoscopy	Heller-Dor	YAN	153	0	0	0	1	2
45	laparoscopy	Heller-Dor	YAM	178	0	0	0	1	2
	laparoscopy	Heller-Dor	ISH	185	0	0	0	1	2
70	laparoscopy	Heller-Dor	TSU	155	0	0	0	1	2
62	laparoscopy	Heller-Dor	OMU	162	0	0	0	1	2
35	laparoscopy	Heller-Dor	KAS	163	0	0	0	1	2
45	laparoscopy	Heller-Dor	YAM	177	0	0	0	1	2
50	laparoscopy	Heller-Dor	ISH	180	0	0	0	1	2
24	laparoscopy	Heller-Dor	YAN	203	0	0	0	1	2
36	laparoscopy	Heller-Dor	TSU	75	0	0	0	1	2
49	laparoscopy	Heller-Dor	YAM	118	0	0	0	1	2
32	laparoscopy	Heller-Dor	OMU	125	0	0	0	1	2
94	laparoscopy	Heller-Dor	KAS	151	0	0	0	1	2
55	laparoscopy	Heller-Dor	ISH	160	0	1	0	1	2
60	laparoscopy	Heller-Dor	YAN	192	0	1	0	1	2
	laparoscopy	Heller-Dor	TSU	80	0	0	0	1	2
62	laparoscopy	Heller-Dor	OMU	146	50	0	0	1	2
50	laparoscopy	Heller-Dor	KAS	153	0	0	0	1	2
34	laparoscopy	Heller-Dor	YAM	162	0	0	0	1	2
65	laparoscopy	Heller-Dor	YAN	162	0	1	0	1	2
48	laparoscopy	Heller-Dor	ISH	195	0	0	0	1	2
75	laparoscopy	Heller-Dor	OMU	108	0	0	0	1	2
32	laparoscopy	Heller-Dor	KAS	124	0	0	0	1	2
62	laparoscopy	Heller-Dor	ISH	210	0	0	0	1	2
79	HALS	Heller-Dor	TSU	230	0	1	0	1	2
45	laparoscopy	Heller-Dor	YAN	237	290		0	1	2
60	laparoscopy	Heller-Dor	TSU	89	0	0	0	1	2
15	laparoscopy	Heller-Dor	YAN	131	0	0	0	1	2
55	laparoscopy	Heller-Dor	OMU	151	0	0	0	1	2
45	laparoscopy	Heller-Dor	ISH	181	0	0	0	1	2
50	laparoscopy	Heller-Dor	KAS	188	50	0	0	1	2
52	laparoscopy	Heller-Dor	TSU	117	0	0	0	1	2
	laparoscopy	Heller-Dor	OMU	132	20	0	0	1	2
50	laparoscopy	Heller-Dor	YAN	156	0		0	1	2
60	laparoscopy	Heller-Dor	ISH	167	2	0	0	1	2
55	laparoscopy	Heller-Dor	KAS	168	0	1	0	1	2
34	laparoscopy	Heller-Dor	TSU	107	0	0	0	1	2
40	laparoscopy	Heller-Dor	OMU	109	0	0	0	1	2
25	laparoscopy	Heller-Dor	KAS	146	0	1	0	1	2
50	laparoscopy	Heller-Dor	YAN	174	0	0	0	1	2
80	laparoscopy	Heller-Dor	OMU	98	0	0	0	1	2
52	laparoscopy	Heller-Dor	TSU	110	0	0	0	1	2
25	laparoscopy	Heller-Dor	YAN	132	0	0	0	1	2
30	laparoscopy	Heller-Dor	KAS	157	0	0	0	1	2
48	laparoscopy	Heller-Dor	KAS	119	0	1	0	1	2
61	laparoscopy	Heller-Dor	TSU	135	0	0	0	1	2
75	laparoscopy	Heller-Dor	YAN	159	0	0	0	1	2
110	laparoscopy	Heller-Dor	OMU	192	0	1	0	1	2
55	laparoscopy	Heller-Dor	TSU	117	0	0	0	1	2
46	laparoscopy	Heller-Dor	YAN	130	0	0	0	1	2
45	laparoscopy	Heller-Dor	KAS	153	50	0	0	1	2
50	laparoscopy	Heller-Dor	OMU	196	0	0	0	1	2
50	laparoscopy	Heller-Dor	TSU	89	0	0	0	1	2
55	laparoscopy	Heller-Dor	KAS	145	0	0	0	1	2
55	laparoscopy	Heller-Dor	OMU	155	0	0	0	1	2
52	laparoscopy	Heller-Dor	YAN	236	75	1	0	1	2
90	laparoscopy	Heller-Dor	TSU	133	0	0	0	1	2
45	laparoscopy	Heller-Dor	KAS	161	0	0	0	1	2
28	laparoscopy	Heller-Dor	OMU	165	0	1	0	1	2
70	laparoscopy	Heller-Dor	YAN	190	50	0	0	1	2
90	laparoscopy	Heller-Dor	TSU	105	100	0	0	1	2
25	laparoscopy	Heller-Dor	YAN	126	0	0	0	1	2
50	laparoscopy	Heller-Dor	OMU	132	0		0	1	2

40	laparoscopy	Heller-Dor	KAS	165	0	0	0	1	2
45	laparoscopy	Heller-Dor	TSU	83	0	0	0	1	2
45	laparoscopy	Heller-Dor·	YAN	128	0	0	0	1	2
60	laparoscopy	Heller-Dor	OMU	150	0	0	0	1	2
55	laparoscopy	Heller-Dor	TSU	100	0	0	0	1	2
55	laparoscopy	Heller-Dor	YAN	121	0	0	0	1	2
20	laparoscopy	Heller-Dor	OMU	163	0	0	0	1	2
40	laparoscopy	Heller-Dor	TSU	67	0	0	0	1	2
50	laparoscopy	Heller-Dor	YAN	119	0	0	0	1	2
40	laparoscopy	Heller-Dor	OMU	229	0	0	0	1	2
49	laparoscopy	Heller-Dor	TSU	91	0	0	0	1	2
	laparoscopy	Heller-Dor	OMU	131	0	0	0	1	2
	laparoscopy	Heller-Dor	YAN	157	0	0	0	1	2
65	laparoscopy	Heller-Dor	TSU	93	0	0	0	1	2
20	laparoscopy	Heller-Dor	OMU	144	0	0	0	1	2
	laparoscopy	Heller-Dor	YAN	148	0	0	0	1	2
75	laparoscopy	Heller-Dor	TSU	137	0	0	0	1	2
	laparoscopy	Heller-Dor	OMU	201	0	0	0	1	2
33	laparoscopy	Heller-Dor	YAN	213	0	0	0	1	2
68	laparoscopy	Heller-Dor	TSU	106	0	0	0	1	2
58	laparoscopy	Heller-Dor	OMU	131	0	1	0	1	2
40	laparoscopy	Heller-Dor	YAN	135	0	0	0	1	2
32	laparoscopy	Heller-Dor	TSU	105	0	0	0	1	2
57	laparoscopy	Heller-Dor	OMU	124	0	0	0	1	2
41	laparoscopy	Heller-Dor	YAN	140	0	0	0	1	2
60	laparoscopy	Heller-Dor	TSU	125	0	0	0	1	2
33	laparoscopy	Heller-Dor	OMU	160	0	0	0	1	2
45	laparoscopy	Heller-Dor	YAN	194	0	0	0	1	2
44	laparoscopy	Heller-Dor	TSU	85	0	0	0	1	2
45	laparoscopy	Heller-Dor	YAN	135	0	0	0	1	2
66	laparoscopy	Heller-Dor	TSU	95	0	0	0	1	2
44	laparoscopy	Heller-Dor	YAN	183	0	0	0	1	2
35	laparoscopy	Heller-Dor	YAN	143	0	0	0	1	2
111.72	laparoscopy	Heller-Dor	TSU	170	0	1	0	2	3
64	laparoscopy	Heller-Dor	TSU	130	0	0	0	1	2
47	laparoscopy	Heller-Dor	YAN	141	0	0	0	1	2
27	laparoscopy	Heller-Dor	YAN	139	0	0	0	1	2
43	laparoscopy	Heller-Dor	YAN	213	30	1	0	1	2
40	laparoscopy	Heller-Dor	YAN	140	0	0	0	1	2
43	laparoscopy	Heller-Dor	YAN	141	0	0	0	1	2
27	laparoscopy	Heller-Dor	YAN	140	0	0	0	1	2

post-operative hospital stay (days)	post-operative patient's satisfaction	GERD recurrent (0=No,1=Yes)
15	5	
7	5	
4	5	0
4	5	0
4	5	0
4		
7	5	0
4	5	0
7	5	1
5	5	0
13	5	0
13	5	0
6		0
12	5	
7	4	0
4		0
9	3	0
6	5	0
4	5	0
11	5	
7		
7		
4	5	0
4	5	0
10	5	0
7	5	
14	5	
4	5	0
4	5	0
17	5	0
4	4	0
7	5	0
7		
4		
13	5	
16	5	0
5	4	1
4	5	0
4	5	0
4	5	1
4		0
9	1	1
8	4	0
4	5	0
4		0
7	5	
7		
6		
10	5	

4		
9	5	0
4	5	
13	4	0
4	5	0
4	5	0
4	5	1
4	5	
4		
4	5	0
6	4	1
5	5	0
4		0
7	5	0
4	5	0
19	5	
4		0
4		0
4	4	0
6	5	0
9	3	
4	3	0
5	5	0
4	5	0
4	5	0
18	5	
6	4	0
4		0
7	5	0
4		
4	5	
4	4	1
10	5	0
4	5	0
7	5	0
8	5	
4	5	0
6	5	1
4	5	0
7		
4		
4	5	0
4	5	
12	5	
4	3	
4	5	1
7	5	0
4	5	0
12	5	1
4	5	0
19	1	0
4	5	0
4	3	0
4	4	1
4	5	0
7	5	0
4		0
4	5	0
4	5	0

4	4	1
4	5	1
4	5	0
8	5	1
8	5	0
7	5	0
4	5	
4	5	0
5	5	1
4	5	
5	2	0
8		
4	5	0
4		
4	5	0
4	4	0
8		
4	5	0
13	3	0
5	5	
10	4	0
4	5	0
5	5	0
15	5	0
5	5	1
4	4	0
5	4	1
6	5	0
4	5	
4		1
4	5	
4	4	0
4	5	
3	5	0
4	4	0
4		
4	5	0
4	4	
4	5	0
10	5	0
7	5	0
4	5	0
4	4	0
4	5	0
4		0
5	5	
4		
6		0
7	5	0
4	5	0
4	5	0
4		0
5	5	0
4	5	0
4	5	
12	5	
4		0
9	5	
4		
4	5	1
4	5	

4	4	1
7		0
9	4	1
4	5	0
4		0
5	5	0
4	5	0
15	4	0
5	5	
6	4	
4	5	0
11		0
4	5	0
4	5	0
3	4	0
13	4	0
4	5	0
6		0
35	4	
4	5	0
4		1
7	5	1
4	4	0
4	5	
4		
4	5	0
4	5	0
9	5	
4	5	
6	5	0
11	5	0
4	5	0
4		0
4	4	0
3		
6		
4	5	1
6	4	0
6	5	0
4	5	0
4	5	0
4	5	0
4	4	
4	4	1
4		
6	5	
6	5	0
4	4	0
4		1
4	5	0
9	5	0
4	5	0
6	5	
4	5	0
7		
4	5	0
5	4	
4	5	1
4		
4	4	0
4	5	1
5	5	0
4	5	0
4		0

12		0
9	5	
4	5	0
4	3	
6	3	
14		0
3		
4		
4	5	0
4	5	0
4	5	
4	4	
4		0
4	5	
9	5	
9	5	0
6	5	
5	5	0
4	5	1
4	4	0
7	4	
4	5	1
4	5	0
4	4	0
4		0
4		
4		
6	5	0
4	5	0
4	5	0
7	5	
7	5	0
4	4	0
4	5	0
5	4	1
5	5	
4	2	0
4		0
3	4	0
17	5	0
5	5	
4	4	1
4	4	0
5	5	1
26	5	1
4	5	1
6		
4	5	0
4		
9	4	0
4	5	0
4	5	0
4	5	1
6	4	
5	4	0
7	5	1
4	5	0
4		1
7	5	0
10	5	0
12	5	0

4		0
6	4	0
7	5	0
9		
6	5	0
7	4	0
9	5	0
4		1
5	5	0
4		0
4	5	0
10	5	
4	5	0
4	5	0
4	5	0
4	5	0
4		
4		
4		0
3		
4	5	0
8	5	0
4	5	1
4	5	0
4		
4		
7		
4	5	0
8		
5	5	0
4	5	0
4	5	0
11	4	0
4	5	
5	3	0
4		
10	4	0
9	5	0
4	5	
4	5	0
4	5	0
4		0
4	5	0
4	5	0
7		1
4	5	1
4		
4		
4	5	0
4	5	
4	5	
4		0
4	4	0
5		
7	5	0
4	5	0
5	4	0
9	5	0
4		
4	5	0
7		
4	3	
4	5	0
4	5	0

4	3	0
4	5	
4		
4	5	0
7	5	1
4	5	0
4	5	0
4		
4	5	
4	5	
4	5	0
4	5	
4	5	0
5	5	0
4		
4	5	0
4	5	1
4	5	0
4	5	0
4		0
4	5	0
5		
4	4	0
4	5	0
4	5	0
6	4	0
4	5	0
4	4	0
4	3	0
4	5	0
4		
4	4	0
4	5	0
7		
4	5	1
4	5	0
4		0
4	4	0
4	5	0
4	3	0
5	5	0
4		
4	5	
4	5	0
4	5	0
4		
4	5	0
4		0
4	5	0
4	5	0
4	5	0
4	5	0
4	5	1
4	5	0
4	5	0
4	5	1
4	5	0
4	4	0
4	5	0
4	5	0
4	5	1
4	5	0
8	5	0
4	5	0

4	5	0
4	5	0
4	4	0
4	5	0
4		0
4	5	0
4	3	0
4	5	0
4	5	
4	4	0
5		0
4	4	1
4	5	
4	5	
4		0
4	5	
4	5	
4		1
4	5	0
4		0
4		
4		0
4		0
4		0
5	5	
6		
4		
4	5	
4		0
4	5	0
4		
4		0
8	5	
4		0
4		1
7		
9	5	
4		
6		
4		
